# Social Determinants of Health, Disaster Vulnerability, Severe and Morbid Obesity in Adults: Triple Jeopardy?

**DOI:** 10.3390/ijerph14121452

**Published:** 2017-11-24

**Authors:** Lesley Gray

**Affiliations:** 1 Department of Primary Health Care & General Practice, Division of Health Sciences, University of Otago, Wellington 6242, New Zealand; lesley.gray@otago.ac.nz; Tel.: +64-(0)21-029-3972; 2 Joint Centre for Disaster Research, School of Psychology, Massey University & GNS Science, Wellington 6140, New Zealand

**Keywords:** disaster risk reduction, disaster vulnerability, social determinants of health, severe and morbid obesity, health inequity

## Abstract

Severe and morbid obesity are associated with highly elevated risks of adverse health outcomes and the prevalence of severe obesity is increasing globally. To date, disaster literature has not considered severe and morbid obesity as a specific vulnerability, despite reports of people being left behind during disasters because of their body size, shape or weight. The complex causes of obesity are associated with the social determinants of health and one’s potential vulnerability to disasters. The absence of appropriate considerations may lead to people being exposed to disproportionate and potentially avoidable risk. The intersection of the social determinants of health, disaster vulnerability, severe and morbid obesity is explored. Previously identified vulnerable groups are also represented in severe and morbid obesity data. This poses the prospect for ‘triple jeopardy’ compounding the social determinants of health, disaster vulnerability and considerations with and for people with morbid obesity. When working to reduce disaster risk for vulnerable groups, the author proposes specific consideration is required to ensure ‘all-of-society engagement and partnership’ in an inclusive, accessible and non-discriminatory manner, to ensure no one is left behind.

## 1. Introduction

The year 2017 may be remembered for a multitude of disasters around the world, not least category five hurricanes Harvey, Irma and Maria devastating Island nations in the Caribbean and the United States of America (USA), deadly earthquakes in Mexico killing hundreds and injuring thousands and some of the worst flooding to hit South Asia in decades. A 500 year flood also engulfed a town in New Zealand following a cyclone causing the Rangitaiki River to burst its banks and evacuations of Islands around the Pacific began in the wake of increased volcanic activity in Indonesia and Vanuatu.

The Sendai Framework for Disaster Risk Reduction 2015–2030 is a global strategy adopted by 187 United Nations (UN) members in Sendai, Japan in 2015 [1]. The Sendai Framework aims to reduce loss of life, livelihood and health from disasters through a range of disaster risk management (DRM) actions. The Sendai Framework built upon the Hyogo Framework for Action [2] which was the global UN plan for disaster risk reduction efforts 2005–2015, including the definition of vulnerability as the conditions determined by “physical, social, economic and environmental factors or processes, which increase the susceptibility of a community to the impact of hazards” [2], (p. 1). This definition of vulnerability aligns closely with the World Health Organization (WHO) definition of the social determinants of health as the “circumstances in which people are born, grow up, live, work and age, and the systems put in place to deal with illness” [3]. These circumstances are shaped by the distribution of money, power and resources at global, national and local levels [4]. Priority one in the Sendai Framework is to understand disaster risk in all its dimensions of vulnerability [1], (p. 36).

Some authors have begun to consider the intersections between disaster vulnerability and the social determinants of health. Lindsay argues that the factors of disaster vulnerability and determinants of health align, presenting the opportunity for shared goals towards identification and management of risks [5]. Plough et al. make the connection between those communities that experience disparities during non-emergency times and the need to build resilience which in turn can strengthen a community’s ability to rally from disasters [6]. Naser-Hall [7] argues for reducing poverty outside of an emergency, so that vulnerable people can address their needs prior to, and in anticipation of, a disaster occurring. Biedrzychi & Koltun [8] provide examples for homeland security and emergency managers to familiarise with the meaning of social determinants in order to integrate into practice and Phibbs et al. introduce the concept of hard-to-reach organisations, rather than hard-to reach populations [9]. Others have specifically considered the intersection of disaster vulnerability and social determinants of health post disaster [10,11], or related to specific vulnerabilities such as end-stage renal disease [12], older persons [13], mortality in tsunami [14], and climate change [15,16]. The prioritization and progress of marginalised communities and populations is key to disaster risk reduction research, with the Sendai Framework [1] aligning with and reaffirming sustainable development goals (SDG) [17], the tenet of SDG being leave no one behind [2].

People with severe or morbid obesity have been negatively impacted in disasters and left behind because of their body size, shape or weight [18,19,20,21,22]. Two illustrative examples are presented here: A patient in New York City was left behind following Superstorm Sandy in 2012 weighing 263 kg and was reportedly too wide for the evacuation sled. This, along with safety concerns for the patient and evacuation personnel, prohibited evacuation down the 15 flights of stairs. Evacuation drills had previously been conducted, although without representative shape and weight in the evacuation sled. This patient’s relative size, shape and weight were reported as the defining factors for her being left behind. This patient was eventually evacuated several days after the storm when power was restored to enable elevators to operate [19]. The second example concerns a person with paraplegia in hospital for a routine procedure who became caught up in the aftermath of Hurricane Katrina in New Orleans (2005). At approximately 380 pounds (172 kg) it is reported that he was deemed too big to be moved for evacuation. Other staff from the facility later stated they did not know he and others were there and they would have attempted to evacuate him [20].

This article presents a perspective on the intersection of the social determinants of health and disaster vulnerability in relation to severe and morbid obesity in adults (aged 18 years and over).

## 2. Severe and Morbid Obesity

The examples presented relate to people who were left behind in relation to their body size, shape and weight. Epidemiological studies have shown substantial increased health risks in people with very high body mass index [23] and people with severe and morbid obesity are disproportionately affected by health consequences of obesity often experiencing premature onset of multiple morbidities [24]. People with severe or morbid obesity can present unique challenges in emergency situations in relation to rescue, evacuation, transport and suitable equipment (medical and shelter) [25,26,27,28] all of which may increase risk of harm.

To date, despite growing references to obesity as a public health epidemic and a raft of policy and intervention efforts to curb it, severe obesity continues to increase in most countries [29,30] and until recently, data for underweight, severe or morbid obesity were not routinely collected to identify global trends [29]. Severe and morbid obesity are classified by body mass index (BMI) which is a proxy for indirectly assessing body fat [31,32], measuring weight adjusted for height. Moderate to severe obesity is classified as BMI ≥ 35.00, and a classification of BMI ≥ 40.00 represents the most significant population health co-morbidity risk group (morbid obesity). Terms commonly applied to BMI of 40 and above in health classifications include morbid obesity, extreme obesity, bariatric, class III obesity [33,34,35,36,37]. Routinely criticised for being a rather blunt instrument, BMI does not distinguish between fat and lean muscle and does not provide information about how fat is distributed throughout the body [34,36,37,38,39,40]. Despite these limitations BMI continues to be the primary index utilised in international medical diagnostic coding and classification and continues as the primary measure for global data on overweight and obesity [29,41].

It is important to acknowledge terms such as morbid obesity are actively contested by academics in the field of fat studies and by supporters of movements such as Health at Every Size^®^ (HAES) [42]. Indeed the term “fat studies” reflects the wish of this discipline to move away from medicalised terms [43]. Whilst acknowledging this debate this paper utilises the terms severe and morbid obesity as the prominent discourse in public health and obesity studies and the direct relationship to classifications referred to in the paper.

## 3. Disaster Vulnerability

According to Twigg [44], vulnerability is the human dimension of disaster. Whilst everyone is potentially put at risk of harm in a disaster, some people due to their circumstances are at greater risk during and following a disaster [45]. These differential circumstances are at the heart of the concept of vulnerability. The characteristics and situation of a person or group are affected by the physical, social, economic and environmental factors or processes [2] creating access blockages or limitations to a variety of different resources. There is increasing evidence that people who have existing vulnerabilities are at greater risk during and following disaster [46,47,48]. More recently there has been a shift in the literature to include positive narratives of people and communities’ capacities for self-protection, adaptation and for mobilized common effort [15,49,50,51].

## 4. Intersecting the Social Determinants of Health and Disaster Vulnerability with Severe or Morbid Obesity

The causes of obesity are complex, involving a combination of influences including but not limited to individual genetics and behaviours [52]. Social, economic and environmental influencers are contributing factors. The type of foods available and whether the environment supports physical activity play a part and these are influenced by our economic situation, education and skills [52,53,54]. Swinburn et al. contend that obesity is a normal response to the obesogenic environment in which we live [53], (p. 804).

### 4.1. Physical and Environmental Dimension of Vulnerability 

Where people live and work are strong determinants for life expectancy as well as vulnerability and disaster risk. Living in seismically active locations or flood prone areas for example. In the USA, Cutter et al. developed an index to map vulnerabilities to environmental hazards [55]. Socio-economic and demographic data were applied to factor spatial patterns. The most vulnerable counties were clustered in south Texas and the Mississippi delta region, both hit hard by major hurricanes in the last 12 years and increasing rain and flooding along the Mississippi delta [55]. Islands in the Pacific Ocean face some of the biggest challenges from natural hazards including but not limited to rising sea levels, volcano and seismic activity. Mexico and New Zealand also face significant natural hazards and these countries and states have some of the highest levels of severe and morbid obesity globally [29].

### 4.2. Economic Dimensions of Vulnerability

Levels of vulnerability are closely linked to the economic status of people, communities and Countries. Being poor is a key driver of exposure to disaster [44,56,57,58,59]. Poor people are more likely to live in areas prone to hazards and be less able to take risk reduction measures [56]. The WHO highlights the most important factors shaping people’s social position to influence the determinants of health include employment and working conditions [60].

Morbid obesity is increasingly associated with lower socio-economic status in both men and women, with a social gradient observed in high-income countries for women [24,61]. The burden of obesity shifts to low socioeconomic status groups and rural areas as a country’s gross domestic product (GDP) increases [62,63]. Income and occupational status influence social inclusion or exclusion of particular groups of people to community life [60]. There is strong evidence that obese employees experience salary penalties and face weight bias in work application and recruitment decisions [64]. Bias and stigma is significantly stronger for obesity and persists compared with many other forms of bias including ethnicity and gender discrimination [65,66]. Obesity bias and stigma pervades workplace employment, health settings, education and general society [64,66,67,68,69]. People with obesity can hold strong biases against people with higher levels of obesity, whereas people in other groups that experience strong biases feel positive towards their group [65].

### 4.3. Social Dimensions of Vulnerability

The social dimensions of vulnerability involve the ability of individuals, groups, organisations or societies to withstand impacts, in this case from disaster. Groups previously identified as being more vulnerable in disasters include:

- people with disabilities (including those with intellectual disability) [45,46,47,48,58,70,71,72,73]
- older people [45,58,74]
- people with severe mental illness [6,75]
- people from culturally and linguistically diverse backgrounds [45,76]
- people with chronic medical conditions [6,10,45]
- children [45,77]
- women [78,79]

The groups identified above have synergies with severe and morbid obesity: Increased weight may be more problematic for people living with disability compared to people with no disability [80]. Associated factors for people with disabilities that may contribute to increasing obesity include lack of accessible environments for physical activity, lack of healthy food choices, medications, pain or energy levels, limited money, transport, social support [81]. In older populations (≥60 years of age) poorer lower extremity mobility is associated with increasing obesity severity in both men and women, with women at increased risk for mobility impairment. Walking, stair climbing, chair rise ability may be especially compromised with severe or morbid obesity [82]. Obesity is multifactorial in persons with serious mental illness, with low socio-economic status contributing to lack of affordable, safe physical activity and limited healthy food choices [83,84,85]. In addition, long term psychotropic medications cause weight gain [86].

People from culturally and linguistically diverse backgrounds face socio-economic inequity. A study by Fothergill et al. in the USA [87] found there are issues specific to race and ethnicity such as culture, language, trust in warning messages and information sources, perceived risk, and level of preparation all contributing to increased vulnerability in disasters [87]. Disproportionate levels of severe and morbid obesity are experienced by different ethnicities and Indigenous populations in countries such as the USA, Mexico, New Zealand, Canada, and Australia [37,88,89,90,91,92]. A very unequal pattern is seen in the prevalence of morbid obesity among adults in New Zealand: in 2011–2013, while the average prevalence of morbid obesity for all adults was 4%, Indigenous Māori prevalence was 7% of males and 12% of females. The prevalence was higher still for Pacific adults in New Zealand (11% of males and 21% of females) [37]. In the USA, morbid obesity levels among non-Hispanic black adults are nearly double those of Hispanic or non-Hispanic whites [93].

There are well documented reports of Indigenous population responses to transition from traditional lifestyle, food supply and physical exertion to “westernised” modern societies [94,95,96]. One such example of an Indigenous population experiencing extreme obesity are the O’odham (Pima Indians of Arizona) [88], representing a living example of such a transition from a traditional lifestyle with limited food supply and high physical activity to a modern, sedentary lifestyle with a consistent energy dense cheap food supply. During and following this transition prevalence of type 2 diabetes and obesity soared to crisis proportions [88]. While it is important to recognise the disproportionate exposure of obesity, Fee [97] points out that ‘race’ and ethnic groupings in relation to obesity are a crude proxy and should not be interpreted to suggest obesity predisposition.

People with morbid obesity are disproportionately affected by health consequences of obesity, often experiencing premature onset of multiple morbidities and are more likely to live with chronic conditions [24,98]. Morbid obesity is stratified across ethnicities and affects more women than men worldwide [24,29].

## 5. Triple Jeopardy?

Maja-Shultz and Swain [70] identified the concept of inter-related vulnerability and the potential for ‘double jeopardy’ during and after disasters. For example, compounded health inequities for already vulnerable people during and after disasters such as limited access or loss of access to usual care [71] or the ability to negotiate service pathways post-disaster [47]. In relation to disaster risk reduction not only do physical, social, economic and environmental factors increase the susceptibility of a community to the impact of hazards, these factors are also the drivers of ill health and contributing factors to severe and morbid obesity. Research on those groups identified as being more vulnerable in disasters is silent on severe or morbid obesity in respect of each of the groups. The presence of severe or morbid obesity delivers ‘triple jeopardy’ adding a further and potentially complex layer of vulnerability that may be caused by or compound pre-existing inequities. For example, women and gender are a priority for action in the social determinants of health because gender inequalities harm the mental and physical health of millions of women globally [99]. Women are already identified as more vulnerable in disasters and make up around half of the global population. The majority of elderly people, children, and disabled people are cared for by women [100,101] and in an emergency the caring role can inhibit a woman’s ability to escape and prevent harm to themselves [79]. Women are more financially vulnerable undertaking unpaid care roles and domestic tasks, with careers that may be interrupted or limited by additional roles. To add further complexity, women are disproportionally represented in severe and morbid obesity statistics and the trend is for more women to be severely obese than underweight by 2025 [29]. To date the disaster literature does not appear to have considered this additional and third layer of jeopardy in disaster risk reduction considerations relating to women. If the adult obesity trend continues severe obesity will overtake underweight in women by 2025 [29].

When looking at the example of the 2009 H1N1 Pandemic, three groups at greatest risk of severe or fatal illness were pregnant women (particularly in the third trimester), children under 2 years of age, and people with chronic conditions of the lungs. Disadvantaged groups were most affected by severe disease and morbid obesity was present in a large percentage of severe and fatal cases. The exact role of obesity is not yet understood as obesity had not been identified as a risk factor in seasonal influenza or previous pandemics [102,103].

## 6. Limitations

The quality of evidence in primary research reports was not appraised and was limited to reports and documents published in English. A limitation of this paper is that it is an individual perspective, based upon personal and professional experiences, key texts and literature, and following five months of field research across the USA, Mexico and Europe. While informed by the current literature, this perspective is not based on a comprehensive systematic literature review and the paper does not provide a detailed analysis of each of the issues as research on disaster risk reduction and severe or morbid obesity is limited thus far. The intention is to widen discussion in the field of disaster risk reduction concerning vulnerable groups.

## 7. Conclusions

Vulnerability to disaster is influenced by many factors including where people are born, how we live, our culture, ethnicity, education and employment. The concept of inter-related vulnerability has been described as a “double jeopardy” [70] and the disproportionate harm or risk that may be faced.

People and communities have tremendous ability to prepare for and adjust to potential hazards if they recognise these and are alerted to specific issues that may be relevant to their circumstances. The Sendai Framework for Disaster Risk Reduction 2015–2030 [1], requires ‘all-of-society engagement and partnership’ in an inclusive, accessible and non-discriminatory manner.

Emergency planners need to widen the current conceptualisation of vulnerability to consider how people with severe and morbid obesity can be included in disaster risk reduction planning and activities. Planners should be cognisant of the extent of bias and stigma concerning obesity and take appropriate steps to mitigate against discrimination in ‘all-of-society engagement and partnership. Research is needed to explore the role of severe and morbid obesity in vulnerability to disaster and to inform risk and risk reduction efforts.

This paper provided a perspective and illustrates ways disparities in health can coalesce and compound each other. Rates of morbid obesity and poor health are over-represented in many countries, regions and communities. Already vulnerable groups face ‘triple jeopardy’ and without appropriate consideration may be exposed to disproportionate and potentially avoidable risk.
